# Expression of *HER-2/neu* in Oral Squamous Cell Carcinoma

**DOI:** 10.31557/APJCP.2020.21.5.1465

**Published:** 2020-05

**Authors:** Sana Mirza, Naila Hadi, Shahid Pervaiz, Sultan Zeb Khan, Sameer A Mokeem, Tariq Abduljabbar, Nawwaf Al-Hamoudi, Fahim Vohra

**Affiliations:** 1 *Department of Oral Pathology, College of Dentistry, Ziauddin Medical University, Karachi, Pakistan. *; 2 *Research and Development, Islamabad Medical and Dental College, Shaheed Zulfiqar Ali Bhutto Medical University, Islamabad, Pakistan. *; 3 *Department of Histopathology and Microbiology, Aga Khan University Hospital, Karachi, Pakistan. *; 4 *Department of Clinical Pathophysiology, Graduate School of Tokyo Dental College, 1-2-2 Masago, Mihama-Ku, Chiba 261-8502, Japan. *; 5 *Department of Periodontics and Community Dentistry, King Saud University, Riyadh, Saudi Arabia.*; 6 *Department of Prosthetic Dental Science, College of Dentistry, King Saud University, Research Chair for Biological Research in Dental Health, Riyadh, Saudi Arabia. *

**Keywords:** Oral squamous cell carcinoma, human epidermal growth factor receptor-2- erbB2, immunohistochemistry

## Abstract

**Background:**

HER-2/neu is a member of the human epidermal growth factor (HER) family of transmembrane tyrosine kinases, which is significantly associated with the pathogenesis of various cancer types. The aim was to evaluate the expression of HER-2/neu in oral squamous cell carcinoma (OSCC) as a potential biomarker to target antigens for specific immunotherapy in OSCC.

**Methods:**

One hundred and forty histologically diagnosed OSCC cases were identified. Four to five-micrometer thick formalin-fixed, paraffin-embedded tumor sections were stained with Haematoxylin and Eosin (H and E). Histological grade was assessed according to WHO/Broders classification, while tumors were staged according to the American Joint Committee on Cancer (AJCC) TNM classification from stage I to IV. Immunohistochemistry was performed by using Rabbit monoclonal antibody against HER-2/neu (EP700Y, cell marquee and diluted 1:50). FISH was performed on positive cases using Vysis PathVysion HER-2 DNA probe (Abbott USA). Probes consist of LSI HER gene spectrum orange and control probe CEP 17 spectrum green.

**Results:**

In this study, males were mostly effected (64.3%) with buccal mucosa (49%) to be the commonly involved site for OSCC. Majority of cases were moderately differentiated (62.1%) and 50.7% tumors were Stage IV. HER-2/neu was found to be positive (2+) in one case of OSCC, however weak to moderate complete membrane staining was observed in >10% of the tumor cells. One hundred and thirty nine cases were HER-2/neu negative. FISH analysis of HER-2/neu positive cases also showed gene amplification (Her2-neu/ CEp 17 = 225/33 = 7.2).

**Conclusions:**

The study showed disparity in the expression of HER-2/neu in OSCC, which is due to multiple reasons. Therefore therapy against HER-2/neu in OSCC is debatable.

## Introduction

The terminology oral cancer is interchangeable with oral squamous cell carcinoma (OSCC) which is estimated to be 90% of all oral neoplasms (Choi and Myers, 2008). Oral cancer is considered as one of the chief public health issue worldwide, accounting for 2-4% of all cancer cases. Despite significant improvements achieved during the last decades in its detection, prevention, and treatment; outcome and prognosis related to OSCC cure and survival is poor due to treatment resistance and tumor recurrence. For most countries, age-adjusted death rates have been estimated to be 3–4 per 100,000 in men and 1.5–2.0 per 100,000 for women (Bray et al., 2018). The mortality rate is steadily even in the younger generation. The removal of two main risk factors: cigarette smoking and alcohol consumption can potentially reduce the incidence of oral cancer up to three-quarters (Beynon et al., 2018). Regardless of the accessibility of oral cavity for clinical examination and advance research into pathogenesis and management, the 5-year survival rate for OSCC has not improved (Mirza et al., 2016). Many molecular markers have been identified reflecting the aggressive behavior of OSCC, however none of these markers have shown unequivocal prognostic or predictive significance (Sharma et al., 2013; Gupta et al., 2016).

The HER-2/neu (ErbB) protein is a tyrosine kinase receptor belonging to tyrosine kinase family comprising of; erbB1, erbB2, erbB3, and erbB4. These proteins play an essential part in cellular growth and differentiation (Mirza et al., 2016; Seifi et al., 2009). The dysregulation of these receptors, lead to an uncontrolled cell cycle, resistance to apoptotic stimuli, invasiveness, chemo-resistance, and angiogenesis (Pardis et al., 2012; Salem et al., 2006). Evidence suggests overexpression of HER-2/neu in advanced diseases, metastasis-associated with poor clinical outcomes in breast and ovarian carcinoma, osteosarcoma; endometrial, salivary, and gastric carcinomas (Bonelloet al., 2018; Khan et al., 2002; Omar et al., 2015). Research studies have correlated the immunohistochemical expression of Her-2/neu with a more aggressive behavior in breast cancer leading to shorter overall survival. Ramic et al., (2013) stated that HER-2/neu is overexpressed in approximately 20-25% of cases of invasive breast cancer (BC), resulting in tumors with more aggressive biological behavior leading to poor prognosis, shorter survival and increased risk of death. 

In a study by Ahmed Al Aziz et al., (2017) it was concluded that Her2/neu can be used as an independent prognostic marker for tumor recurrence after complete resection of gastrointestinal stromal tumors. Concerning oral squamous cell carcinoma, the biological behavior of tumor is highly variable and studies on HER-2/neu are discordant and insufficient (Dragomir et al., 2012; Kouhsoltani et al., 2015; Schartinger et al., 2009). The incidence of Her2/ neu overexpression has ranged from 0 to 40% (Angiero et al., 2008; Cavalot et al., 2007; Sardari et al., 2012). By contrast, Singla et al., (2018) reported Her-2/neu expression to be negative, though cytoplasmic positivity was observed in a few cases. Continued therapeutic developments have resulted in targeted therapies against HER-2/neu in the breast, gastric and lung carcinomas (Liu et al., 2010; Moelanset al., 2011). Bang et al conducted a phase 3, randomized controlled trial and concluded that trastuzumab in combination with chemotherapy exhibits both efficacy and safety for the first-line treatment of advanced gastric cancer with expression of HER-2 (Bang et al., 2010). In a similar report, Ohashi et al., (2017) reported that approximately 5% of NSCLC tumors possess HER2 alterations, and Trastuzumab emtansine (an anti-HER2 antibody conjugated with a vinca alkaloid) has shown excellent antitumor effects against HER2/neu. The therapy targeting this pathway in OSCC could uncover valuable evidence, and a clinical trial about this signal would be worthy. Therefore, the present study aimed to evaluate the expression of HER-2/neu in OSCC to identify a potential biomarker, potentially used as a target antigen for specific immunotherapy against OSCC.

## Materials and Methods

This study included 140 cases of Oral Squamous Cell Carcinoma (OSCC) presenting to the maxillofacial department of Ziauddin college of Dentistry. The processing was performed at Histopathology Laboratory of Ziauddin Hospital, Karachi, Pakistan. The clinic-pathologic data of each case was collected using a questionnaire. Cases without complete clinicopathologic data, inadequate paraffin-embedded material, incisional biopsies, and recurrent OSCC were excluded. Specimens were routinely processed, fixed overnight in 10% buffered formalin and were examined grossly according to the standard guidelines. Four to five-micrometer thick formalin-fixed, paraffin-embedded tumor sections were stained with H and E. Histological grade was assessed according to WHO/Broders classification (El-Mofty, 2007; Schwab, 2011). Tumors were staged according to the American Joint Committee on Cancer (AJCC) TNM classification seventh edition from stage I–IV. The ethics committee of Ziauddin University, College of Dentistry, provided ethical approval.

*Immunohistochemistry evaluation*

Immunohistochemistry was performed by using Rabbit monoclonal antibody against HER-2/Neu (EP700Y, cell marquee and diluted 1:50). The HER-2 protein immune-expression was analyzed using the American Society of Clinical Oncology/College of American Pathologists (ASCO/CAP) guidelines for Her-2 testing in Breast Cancer, as shown below (Bernardes et al., 2011).

‘0’- no staining or membrane staining is observed in < 10% of the tumor cells;

‘1+’- faint/barely perceivable membrane staining is detected in > 10% of the tumor cells, and only part of the membrane is stained;

‘2+’-weak to moderate complete membrane staining is observed in >10% of the tumor cells; 

‘3+’-strong complete membrane staining is observed in > 30% of the tumor cells.

*Fluorescence in situ hybridization*

A 4-5-micron paraffin-embedded tissue section was taken on a positively charged slide, the sample was pretreated with xylene to remove paraffin and ethanol then subjected to proteinase treatment (pepsin) before proceeding to apply FISH probe. Vysis PathVysion HER-2 DNA probe (Abbott USA) was used. Probes consisted of LSI HER gene spectrum orange and control probe CEP 17 spectrum green. The probe is applied to the slide and placed in thermobrite for denaturation at 73°C for 7 minutes and hybridization for 24hrs. Post washing was performed after 24hrs with a reagent NP-40 to remove the unattached probe and apply Dapi (counterstain). On fluorescent microscopy, approximately 20 nuclei were counted with a red signal representing the HER-2 gene and green signal CEP 17.

*Interpretation of FISH*

FISH was analyzed using the American Society of Clinical Oncology/College of American Pathologists (ASCO/CAP) guidelines for Her-2 testing in breast cancer as shown below (Richardson et al., 2012; Wolff et al., 2007).

Negative (not amplified) - Average Her2 copy number < 4.0 signals/ cell.

Equivocal – Average Her2 copy number ≥ 4.0 and < 6.0 signals/ cell.

Positive (amplified) – Average Her2 copy number ≥ 6.0 signals/cell.

*Statistical analysis*

Statistical analysis was performed using the SPSS software program (v.21.0 for Windows, IBM, Chicago, IL). Data for categorical variables such as age, gender, lymph node involvement, site of the primary lesion, perineural invasion (PNI), lymphovascular invasion (LVI), distant metastasis (DM), and stage of the tumor were expressed in frequencies and percentages. A chi-square test was used among different groups to construct association. Since tumor size and tumor thickness were continuous variables so, Kruskal-Willis and Mann-Whitney test were used to find the difference in the mean ranks of tumor size and thickness. A p-value of less than 0.05 was regarded as statistically significant.

## Results

This cross-sectional study was carried out on 140 OSCC patients, with 90 (64.3%) males and 50 (35.7%) females. Most of the patients (41; 29.3 %) age ranged between 49 to 58 years. Buccal mucosa was found to be the most common site (69; 49.3%), followed by mandible and tongue (24; 17.1%). Most of the tumors were moderately differentiated (87; 62.1%) followed by poorly differentiated (30; 21.3%) and (23; 17.1%) well-differentiated OSCC. Maximum number of patients had stage IV tumors (71; 50.7%), followed by stage II (35; 25.0), stage III (24; 17.1%) and stage I (10; 7.1%) tumors. Lymph node metastasis was present in 51.4% (n = 72) and absent in 48.5% (n=68) of OSCC patients. The mean tumor size was 3.4 ± 1.9 (range 0.5-11cm) with the mean tumor thickness of 1.8 mm (range 0.2-11mm). Perineural invasion was present in 22.9% and absent in 77.1% of OSCC patients. While the lymphovascular invasion was present in 8.6% and absent in 91.4% of OSCC patients. Distant metastases (Stage IVC) was found in only 8 (5.7%) OSCC patients.

**Figure 1:**
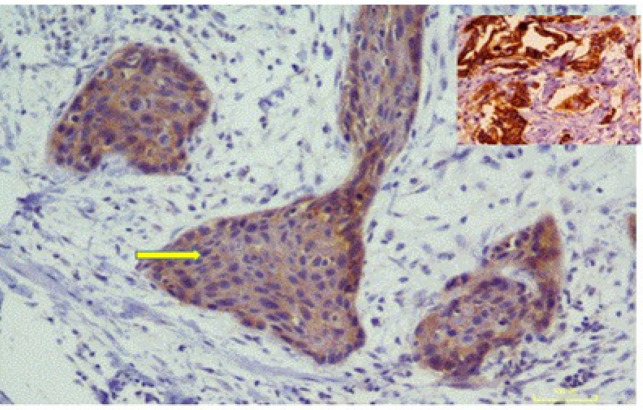
Immunohistochemical Staining for OSCC- Showing Weak to Moderate Dark Brown Membranous Staining (arrow) of HER-2/neu (2+) 200X. Inset in shows positive control for Her2/neu expression (invasive ductal carcinoma of breast, 400X). (Case. No. 90)

**Figure 2:**
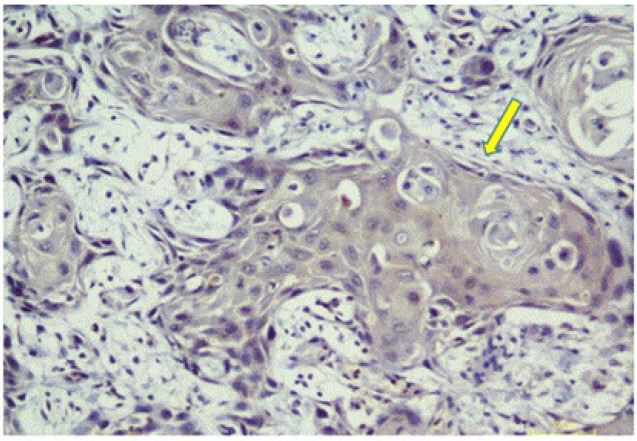
Immunohistochemical Expression of HER-2/neu in OSCC Showing Negative Expression (200X) (arrows) (Case. No.68).

**Figure 3:**
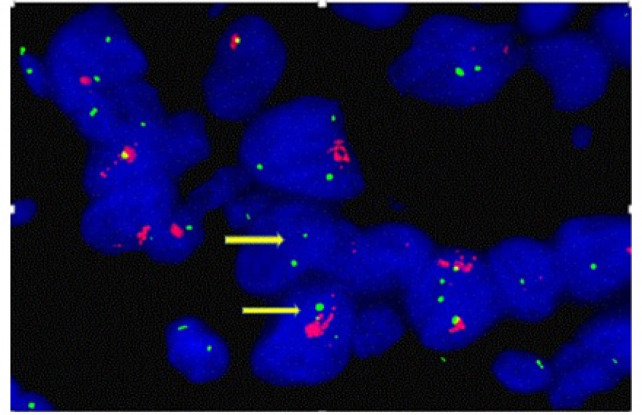
Representative FISH Image of OSCC Showing Interphase Index Using HER-2/CEP 17 Dual-Color Probe. Her2 gene amplification is seen. Gene signals are red- labeled, CEP 17 signals are green-labeled (arrow) (Case. No.90).

HER-2/neu was found to be positive (2+) in one case of OSCC, with weak to moderate complete membrane staining observed in >10% of the tumor cells (Figure 1). The remaining 139 cases were HER-2/neu negative (Figure 2). FISH analysis of Her2/neu positive case also showed gene amplification (HER2-neu/ CEp 17 = 225/33 = 7.2) (Figure 3).

## Discussion

The immunohistochemical expression of Her2/neu in oral cancer has shown wide variation (3-34%) (Bei et al., 2004; Roberto Bei et al., 2001; Chen et al., 2003). This disparity in results regarding the HER-2/neu overexpression is related to clinicopathological parameters of OSCC (Craven et al., 1992; Field et al., 1992; Pardis et al., 2012; Rivière, Becker, and Löning, 1991). Hence the role of HER-2/neu in carcinogenesis of oral squamous cell carcinoma remains controversial. The present study aimed to evaluate the expression of HER-2/neu in a subset of South-East Asian population as data is scarce in such OSCC patients. In the present study, only one case (0.72%) showed overexpression (2+) with 139 (99.28%) cases showing no significant staining of cancer tissue. This is similar to the findings of previous studies in discrete populations reporting no overexpression of HER-2/neu in OSCC (Angiero et al., 2008; Ekberg et al., 2005; Pardis et al., 2012; Sardari et al., 2012; Shintani et al., 2004). On the other hand, some studies have reported the overexpression of HER-2/neu suggesting its use as a marker to differentiate healthy from cancer tissues (Cavalot et al., 2007; Fonget al., 2008; Lebeau et al., 2001). On reviewing the current evidence, it is hypothesized that overexpression of HER-2/neu occurs only in a small number of OSCC cases. To support our conclusion, we carried out an in-depth analysis of different studies by recording the total number of cases studied, the number of cases showing positive outcomes and intensity of positive expression (1+, 2+, 3+ and 4+). According to CAP guidelines for HER-2/neu immunostaining, an outcome of 3+ is regarded as overexpression of protein (Richardson et al., 2012; Wolff et al., 2007). Many of the authors including Chen et al., (2003) reported overexpression of HER-2/neu in 41% (n=59) cases, however failed to mention the scores. Similarly, Azemar et al., (2000) reported the overexpression of HER-2/neu in 58% of cases (n=45) but did not report the scores. On the other hand, Fong et al (33) reported HER-2/neu overexpression in 50% of his cases (12 cases 1+, and 3 cases 2+), while Seifi et al., (2009) reported overexpression of HER-2/neu in 72% of OSCC cases (5 cases 1+, 8 cases 2+). However, in both these studies, none of the cases were 3+ which is regarded as overexpression. Dragomi et al., (2012) reported five cases as 3+ (n= 44) while Schartinger et al., (2009) (n=116), (Bernardes, et al., 2010) (n=46) and Khan et al., (2002) (n=26) reported only one case with 3+ overexpression. 

In a study by Wilkman et al., (1998) only 11 cases of squamous cell carcinoma reported intense staining of her2/neu in the poorly differentiated epithelium. While in the present study, no difference in expression of HER-2/neu was seen among different histological grades of OSCC (p=0.23). Beckhardt et al., (1995) revealed only 6 of 38 (16%) OSCC tissue sections with HER-2 oncoprotein overexpression. While (Stoicanescu et al., 2013) reported overexpression of HER-2/neu in only 25% of cases among which, 5.2% were 1+, 14.7% were 2+ and only four cases had 3+ expression. Hanken et al., (2014) reported overexpression of HER-2/neu in only 4 cases out of 196 (1 case 1+, 2 cases 2+ and 1 case 3+) . They also carried our FISH analysis reporting gene amplification in 6 out of 207 cases, suggesting that these cases can benefit from antiHER-2 therapy. Birkeland et al., (2016) reported overexpression in only 2 out of 92 cases of OSCC. Both the authors concluded that despite this low Her2/neu overexpression of only 1 – 2%, those few patients could benefit from anti HER-2 therapy, thus highlighting the role of this receptor in personalized medicine.

In multiple studies conducted to study HER-2/neu expression in OSCC, purely membranous staining (Craven et al., 1992; Hoffmann et al., 2001; Khademi et al., 2002) or cytoplasmic staining (Angiero et al., 2008; Craven et al., 1992; Field et al., 1992; Khademi et al., 2002), and mixed membranous-cytoplasmic staining (Bei et al., 2004; Craven et al., 1992; Ibrahimet al., 1997; Rautava et al., 2008; Xia et al., 1997; Xia et al., 1999) have been reported. In squamous cell carcinoma, cytoplasmic staining is commonly reported but according to Cavalot et al., (2007) it is said to be a technical artifact due to cross-reactive antibodies possibly with keratin or during antigen retrieval process. For better, authentic and more reliable results we considered purely membranous staining as positive for the cases in the present study.

Literature has shown inconsistency among correlation of HER-2/neu with clinicopathological parameters of oral squamous cell carcinoma irrespective of overexpression of HER-2/neu. In the present study, there was no association of HER-2/neu expression with any of the clinicopathological parameters. This is similar to other studies (Cavalot et al., 2007; Khan et al., 2002) with no significant association between HER-2/neu positivity with age, sex, race, tumor site, tumor stage, grade, and recurrence. A study by Fong et al., (2008) reported overexpression of HER-2/neu in stage IV OSCC as compared to stage I-III OSCC cases. This is in contrasts with the present study showing no significant association with tumor stage (p=0.388). Therefore, researchers like Riviere et al., (1991), Field et al., (1992), Khan et al., (2002), Ekberg et al., (2005) and Angiero et al., (2008) did not suggest HER-2/neu as a prognostic factor or treatment indicator in OSCC patients. A possible explanation for disparity among study outcomes is a lack of standardization of the assay methods (the direct, indirect technique of immunohistochemistry) and use of different techniques (immunosorbent assay, radioimmunoassay, IHC). A careful review of studies showed a variation in the type of antibody used that recognize different epitopes of protein (Clone CerbB2, CB11, ICR1b, polyclonal DAKO, monoclonal enzyme), in addition to lack of specific criteria for positive staining of HER2/neu protein (membrane and/ or cytoplasmic). In particular, the significance of cytoplasmic staining is the most widely debated question. Gender variation and differences in the site of primary tumors are also considered to contribute to this disparity in results (Cavalot et al., 2007).

The present study supports the idea that, despite low Her2/neu overexpression (1-2 %), patients with positive results could benefit from anti-cancer/anti-Her2 therapy. As oral cavity is part of gastrointestinal tract, Her2 testing on gastric biopsies should be investigated and optimized for immunohistochemical scoring in OSCC tissues employing future studies. Further studies are recommended to establish standard immunohistochemistry staining protocols to aid uniform reporting of protooncogene in OSCC.

In conclusion, the study showed disparity in the expression of HER-2/neu in OSCC which is due to multiple reasons. Therefore therapy against HER-2/neu in OSCC is debatable. 
